# Phosphoproteome-Entailed Kinase–Substrate Landscape of Human–DENV-2 Interaction

**DOI:** 10.3390/ijms27062718

**Published:** 2026-03-17

**Authors:** Ayisha A. Jabbar, Vineetha Shaji, Akash Anil, Mahammad Nisar, Sowmya Soman, Ganesh Prasad, Chandran S. Abhinand, Prashant Kumar Modi, Madathiparambil Gopalakrishnan Madanan, Abhithaj Jayanandan, Rajendra Pilankatta, Rajesh Raju

**Affiliations:** 1 Centre for Integrative Omics Data Science (CIODS), Yenepoya (Deemed to Be University), Mangalore 575018, Karnataka, India; ayishaajabbar.ciods@yenepoya.edu.in (A.A.J.); vineethashaji@yenepoya.edu.in (V.S.); akasha.ciods@yenepoya.edu.in (A.A.); nisar.ciods@yenepoya.edu.in (M.N.); sowmyasoman.ciods@yenepoya.edu.in (S.S.); abhinandcs@iav.res.in (C.S.A.); abhithaj.j.ciods@yenepoya.edu.in (A.J.); 2 Centre for Systems Biology and Molecular Medicine (CSBMM), Yenepoya Research Centre, Yenepoya (Deemed to Be University), Mangalore 575018, Karnataka, India; prashantmodi@yenepoya.edu.in; 3 Department of Biochemistry, Yenepoya Medical College, Yenepoya (Deemed to Be University), Mangalore 575018, Karnataka, India; ganesh@yenepoya.edu.in; 4 Department of Virus Genomics, Bioinformatics, and Statistics, Institute of Advanced Virology (IAV), Bio 360 Life Sciences Park, Thonnakkal, Thiruvananthapuram 695317, Kerala, India; 5 ICMR—Regional Medical Research Centre, Port Blair 744103, Andaman and Nicobar Islands, India; madananmg@gmail.com; 6 Department of Biochemistry, Central University of Kerala, Kasaragod 671316, Kerala, India; praj74@cukerala.ac.in

**Keywords:** dengue virus, kinase–substrate phosphomotifs, phosphorylation, protein–protein interaction, molecular docking

## Abstract

Dengue virus (DENV) is a mosquito-borne RNA virus that causes serious illness in humans, ranging from mild fever to severe clinical manifestations, with dengue virus type 2 (DENV-2) being the most virulent among its four serotypes. Despite extensive research, no specific antiviral therapy is currently available, making the host-directed method an appealing therapeutic approach. Evidence shows that DENV manipulates host kinase-driven phosphorylation pathways to control viral pathogenesis. Using the kinase–substrate phosphomotif approach, we predicted phosphorylation sites across the DENV proteome and their potential human kinases. The predicted kinase–substrate interactions were systematically integrated with DENV-2-induced human phosphoproteome datasets, protein–protein interactions, and experimentally-validated viral phosphosites. The therapeutic relevance of the identified host kinases was corroborated by the impact of their inhibitors on DENV-2 infection. Among the 359 potential human kinases predicted to phosphorylate DENV-2 proteins, based on human phosphoproteome and kinase–viral protein interaction analyses, CDK9 emerged as a central hub kinase. Molecular docking analyses further revealed that the host kinases CDK9, EEF2K, HASPIN, and TNNI3K form stable interactions with the viral capsid and NS5 proteins. Additionally, a conservation analysis suggested that the predicted phosphorylation sites are evolutionarily conserved across DENV-2 strains. Computational prediction tools supported the predicted kinase–substrate interactions, underscoring the role of host kinases as key regulators of DENV infection, which may act as potential therapeutic targets. This study highlights the interplay between dengue viral and host proteins, providing insights into host-directed therapeutic strategies for DENV-2 infection and their potential to address the current lack of effective antiviral interventions.

## 1. Introduction

The dengue virus (DENV) is a mosquito-transmitted flavivirus that poses a serious threat to public health in tropical and subtropical regions worldwide. DENV affects nearly 3 billion people worldwide with a mortality rate of about 20% [1]. Dengue fever, often referred to as ‘breakbone fever’ or ‘seven-day fever’, is characterized by severe joint pain, muscle cramps, and a sudden spike in temperature, reflecting the severity of the infection and its symptomatic course, typically lasting for more than a week [2]. It is mainly transmitted through the bite of Aedes mosquitoes, which serve as the primary vectors of the virus. The virus exists in four serotypes (DENV-1, DENV-2, DENV-3, and DENV-4) [3]. According to the WHO 2009 classification, dengue is grouped into dengue without warning signs, dengue with warning signs (including abdominal pain, persistent vomiting, fluid accumulation, mucosal bleeding, lethargy, liver enlargement, and rising hematocrit with declining platelet counts), and severe dengue, which involves severe plasma leakage, major bleeding, or organ dysfunction [4].

DENV possesses a single-stranded RNA genome of approximately 11 kilobases in length. Once it enters the host cell, the viral genome is directly translated by the host’s ribosomes and protein synthesis machinery into a single polyprotein, which is subsequently cleaved into ten distinct proteins. These include three structural proteins (capsid (C), precursor membrane/membrane (prM/M), and envelope (E)), and seven non-structural proteins (NS1, NS2A, NS2B, NS3, NS4A, NS4B, and NS5). The structural proteins are essential for viral attachment, entry, assembly, and secretion, while the non-structural proteins contribute to viral replication, immune evasion, and various enzymatic functions crucial for the viral life cycle [5]. The envelope protein facilitates viral entry into the host cell [6]. The capsid protein mainly functions to enclose the viral RNA during assembly and then release it into the host cell during infection [7]. Membrane proteins form ion channels to facilitate viral release from the host cells, playing a critical role in viral entry and replication [8]. Non-structural protein NS1 interacts with NS4A/B to increase replication, NS3 acts as a helicase and protease, NS4A triggers autophagy, and NS4B helps release NS3 from viral RNA. NS5 functions both as an RNA-dependent RNA polymerase and a methyltransferase, which are essential for viral replication and polyprotein translation, making it a significant target for vaccine development and antiviral therapies [9].

Currently, dengue infection lacks a specific antiviral treatment, and management primarily focuses on alleviating symptoms. The development of a vaccine that protects against all four DENV strains is a primary goal set by the World Health Organization (WHO). However, developing an effective vaccine is challenging owing to the limited knowledge of viral pathogenesis [10]. Small molecules that inhibit different stages of DENV replication can help researchers better understand the mechanisms of virus–host interactions and may also aid in the development of therapeutic strategies against DENV infection [11]. In recent studies, our group has employed molecular docking and molecular dynamics simulations to identify potential inhibitors targeting specific viral proteins involved in infection and replication [12,13,14,15,16]. Host kinase inhibitors are promising candidates for antiviral drug development, as viruses hijack multiple host kinases at different stages of their lifecycle [17]. DENV infection is known to activate specific signaling molecules within the mitogen-activated protein kinase [MAPK] pathway, such as c-Jun N-terminal kinase [JNK], p38, neurotrophic receptor tyrosine kinase 1 [NTRK1], MAP kinase-activated protein kinase 5 [MAPKAPK5], and c-Src/FYN [18]. Most cell signaling pathways rely on a series of phosphorylation steps, which means kinases play a key role and can be targeted to treat many diseases caused by abnormal regulation [19].

Recent studies suggest that compounds targeting host cellular pathways may help control DENV infection [11]. This therapeutic strategy has already shown effectiveness against other viral infections, including human papillomavirus, hepatitis B and C viruses, and human immunodeficiency virus [HIV] [20]. DENV proteins have been shown to interact with host proteins. Many of these interactions are physiologically relevant and involve post-translational modifications, including phosphorylation, which influence viral and host protein function [21]. Emerging evidence indicates that host kinases play a significant role in DENV infection and pathogenesis supporting viral entry, enhancing genome replication and protein synthesis, integrating virion formation, and promoting viral release [22]. Phosphorylation of the DENV protein NS1 by host kinases can modulate its structural conformation and functional properties, influencing viral replication and assembly [23]. Similarly, phosphorylation of the DENV protein NS5 is known to influence its subcellular localization and possibly its function. To explore the role of these kinases in viral replication, various kinase inhibitors were tested and were shown to interfere with specific stages of the dengue virus life cycle, leading to significant reductions in viral levels [11].

Since phosphorylation plays a vital role in modulating protein function, identifying phosphorylation sites on DENV protein and their corresponding kinase is crucial for understanding the mechanisms of viral replication and disease progression. Kinases usually recognize and phosphorylate target proteins that contain specific amino acid sequences known as phosphorylation motifs. These sequence patterns are commonly used in computational approaches to predict potential kinase–substrate interactions [24]. Johnson et al. (2022) and Yaron-Barir et al. (2024) generated highly specific substrate specificities for serine/threonine and tyrosine kinases using synthetic peptide libraries and positional scanning peptide arrays [25,26]. The phosphomotif patterns for 384 human kinases, generated by Poll B. G. et al. (2024), using the large-scale phosphorylation data from Sugiyama N. et al.’s study (2019), serve as a recognition pattern to predict potential kinase–substrate interactions [24,27]. In this study, phosphorylation sites on dengue viral proteins were computationally mapped to their corresponding upstream human kinases to elucidate key host–pathogen interactions. This integrative approach provides valuable insights into how host signaling pathways modulate viral replication and underscores the potential of targeting these kinases as promising strategies for future antiviral therapy.

## 2. Results

### 2.1. Computational Identification of Kinase–Target Motifs in DENV Proteome

We retrieved 32,652 protein sequence entries for different DENV types and strains from the UniProt database. Pattern motif analysis of these sequences, based on the methods of Johnson et al. (2022) and Yaron-Barir et al. (2024) [25,26], identified 351 potential kinases targeting 27,676 variant sequences across the 10 DENV proteins. Among these kinases, 284 were serine/threonine kinases, 45 were tyrosine kinases, and 22 were dual-specificity kinases. All 351 kinases were also predicted in the DENV-2 sequences.

**Figure 1:**
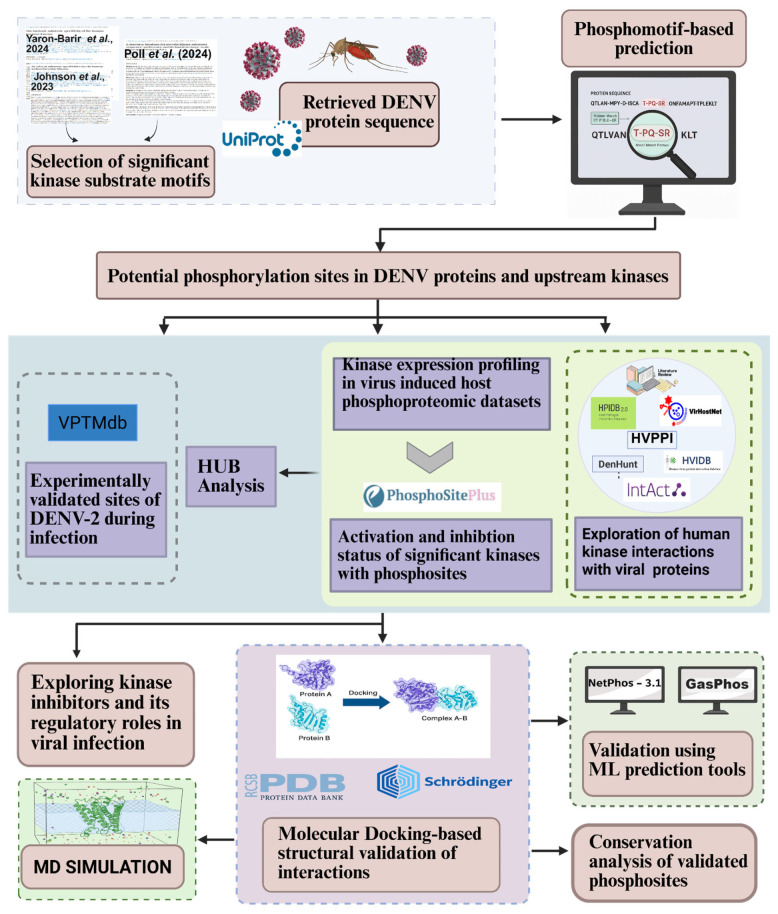
Workflow of the integrative analysis used to identify host kinases potentially phosphorylating DENV-2 proteins. References cited from [25,26,27].

Unlike the Johnson et al. (2022) and Yaron-Barir et al. (2024) [25,26] pattern motifs, the Poll B.G. et al. (2024) [27] motif contained variable ‘X’ positions that can match with any amino acid, resulting in a more flexible motif structure and a broader range of potential kinase–substrate matches. Using this pattern, 68 kinases were predicted to potentially target 32,444 variant sequences, of which 35 were serine/threonine kinases, 25 were tyrosine kinases, and eight were dual-specificity kinases. Among these, 49 kinases were identified with the potential to phosphorylate DENV-2 proteins. A detailed summary of the results is provided in Supplementary Tables S2 and S3, and the complete analysis workflow is shown in Figure 1.

### 2.2. Phosphoproteomic Evidence of Kinase Phosphosite Regulation in DENV-2 Infection

Based on available phosphoproteomic data from DENV-2-infected human K562 cells [28], we identified host kinases showing differential phosphorylation patterns associated with viral infection. Kinase motif pattern analysis, based on the studies by Johnson et al. (2022) and Yaron-Barir et al. (2024) [25,26], identified 38 kinases that overlapped with the phosphoproteomics dataset, indicating their potential functional roles in DENV-2 infection (Supplementary Table S4). Among these, mitogen-activated protein kinase kinase kinase 7 [MAP3K7]-S439, microtubule affinity-regulating kinase 3 [MARK3]-S390, MARK3-S391, DNA-dependent protein kinase catalytic subunit [PRKDC]-S2612, protein kinase C theta [PRKCQ]-S695, microtubule affinity-regulating kinase 2 [MARK2]-S40, cyclin-dependent kinase 1 [CDK1]-T14, CDK9-T186, cyclin-dependent kinase 13 [CDK13]-T871, α-protein kinase 3 [ALPK3]-T946, and cyclin-dependent kinase 12 [CDK12]-T893 were found to be upregulated, whereas CDK1-Y15 and mitogen-activated protein kinase kinase 2 [MAP2K2]-S295, and EEF2K-S18 were downregulated under infected conditions. Based on the regulatory information from PhosphoSitePlus indicated that CDK12-T893, CDK9-T186, and PRKCQ-S695 are kinase activation associated phosphosites. In contrast, the phosphosites, CDK1-T14 and CDK1-Y15 were kinase activity inhibition associated phosphosites. 

An analysis by Poll B. G. et al. (2024) [27] indicated that CDK9 and EEF2K each carried a distinct regulatory phosphosite (CDK9-T186 and EEF2K-S18), both of which were also experimentally shown to be modulated during DENV-2 infection in the phosphoproteomics dataset. From this dataset, we found that CDK9-T186 was upregulated, while EEF2K-S18 expression was downregulated under DENV-2 infection conditions. As noted above, CDK9-T186 is an activation site. We consider these phosphorylation sites to be part of a dynamic regulatory mechanism involving host kinases during DENV-2 infection. The upregulation of activation sites and the downregulation of inhibitory sites result in enhanced kinase activity. While our findings indicate that these kinases may play a potential role in modulating host responses or supporting viral processes, further experimental validation is needed to confirm their function, making these results promising leads for future studies to uncover the mechanistic role of phosphorylation in DENV-2 pathogenesis.

### 2.3. Identification of DENV Phosphosites and Associated Human Kinase Predictions

To further substantiate our predictions of host kinase and DENV-2 protein interactions, we analyzed experimentally validated phosphosite data of DENV-2 proteins to identify candidate upstream host kinases responsible for their phosphorylation. DENV-2 NS5 was identified as the phosphorylated protein with phosphorylation sites T395 [29] and T449 [30] from the VPTMdb database [31]. Casein kinase 2 [CK2] was predicted to phosphorylate T395, while cGMP-dependent protein kinase [PKG] was associated with phosphorylation at T449. Only two phosphorylation sites have been experimentally validated in the DENV infection-based studies, representing a major limitation in the available dengue-specific phosphosite data. The highly specific kinase–substrate motif datasets of Johnson et al. (2022) and Yaron-Barir et al. (2024) [25,26] did not identify candidate kinases for these particular viral phosphosites, as the corresponding +3/−3 phosphomotif sequences (RMCTREE and KCETCVY) were not represented within the studied motifs. However, analysis based on the Poll B. G. et al. (2024) [27] motif patterns predicted phosphorylation at these same sites but by a different set of kinases: activin receptor type I [ACVR1], bone morphogenetic protein receptor type-1A [BMPR1A], and microtubule-associated serine/threonine kinase 2 [MAST2] were predicted to target T395, while TNNI3K was associated with T449. These host kinases are thought to play a critical role in phosphorylation of DENV, particularly on the NS5 protein, which is essential for viral replication. NS5 functions as both RNA-dependent RNA polymerase and RNA methyltransferase, facilitating viral RNA synthesis and capping to protect the genome and support polyprotein translation [9]. Therefore, phosphorylation of NS5 at these specific sites could be essential for regulating its enzymatic activity during infection.

### 2.4. Interaction Analysis of Human Kinases and DENV-2 Proteins

The virus exploits host cellular machinery for replication by forming specific interactions with host proteins. Human–viral protein interaction analysis identified 42 host kinases, predicted from motif-based analyses by Johnson et al. (2022) and Yaron-Barir et al. (2024) [25,26], that potentially interact with the DENV-2 genome polyprotein. Notably, among the predicted kinases, PRKDC, pre-mRNA processing factor 4 kinase [PRP4K], CDK1, MAP2K2, checkpoint kinase 1 [CHEK1], RAF proto-oncogene serine/threonine protein kinase [RAF1], RAC-alpha serine/threonine-protein kinase [AKT1], serine/arginine-rich protein kinase-1 [SRPK1], and bone morphogenetic protein 2-inducible kinase [BMP2K] were also detected in the DENV-2.infected phosphoproteomic datasets, further reinforcing their involvement in DENV-2 infection.

**Figure 2:**
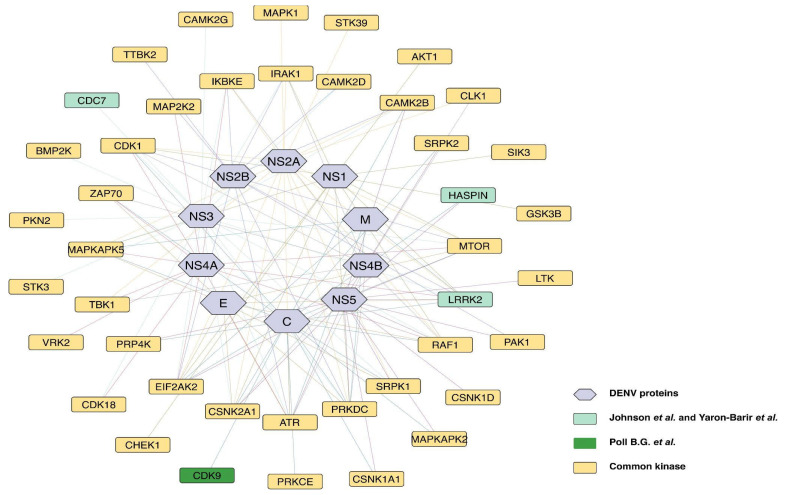
DENV-2 proteins and their predicted human kinase interactions derived from motif-based analyses. The network demonstrates interactions between DENV-2 proteins (shown in purple) and human kinases predicted through motif pattern analysis. Colored connecting lines represent the interaction links corresponding to each individual DENV-2 protein in the network. Kinases highlighted in light green represent those uniquely predicted by the motif pattern analyses of Johnson et al. and Yaron-Barir et al. [25,26]; kinases shown in dark green represent those predicted only by the Poll B. G. et al. [27] motif model. Kinases represented in cream indicate the common set identified by Poll B. G. et al., Johnson et al. and Yaron-Barir et al.

Similarly, analysis based on the Poll B. G. et al. (2024) [27] motif patterns identified four kinases, leucine-rich repeat kinase 2 [LRRK2], CDK9, HASPIN, and cell division cycle 7 [CDC7] that showed interaction with DENV-2 genome polyprotein. Among these, CDK9 was previously reported in human phosphoproteomics datasets to exhibit modulated phosphorylation during DENV-2 infection, suggesting a possible functional role in the viral life cycle. Furthermore, prior studies have reported the role of CDK9 in DENV infection [20]. These interactions between host kinases and DENV-2 proteins strongly support our prediction that DENV-2 exploits host kinase networks to facilitate its infection cycle. A summary of these kinase–protein interactions is shown in Figure 2, and the detailed information is in Table 1.

### 2.5. Network-Based Identification of Hub Kinase

**Figure 3:**
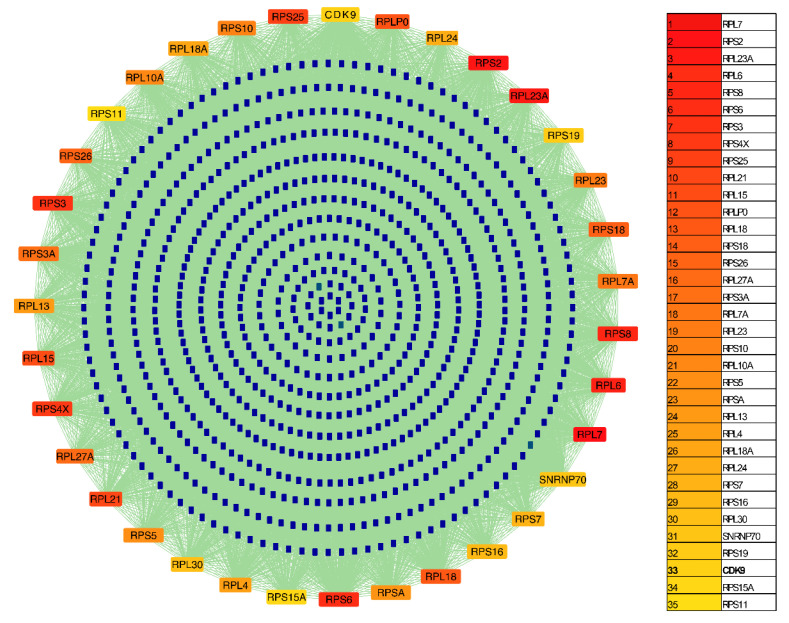
Integrated dengue-associated interaction network showing the top 35 ranked proteins. Nodes of different colors in the outer circle represent the top 35 proteins. The dark blue nodes in the inner region represent their interacting proteins, and the lines represent the interaction between nodes.

Hub analysis using the MCC algorithm in CytoHubba ranked 35 high-confidence proteins based on their network connectivity within the integrated interaction network (Figure 3). The network comprised 888 nodes and 31,115 edges, representing DENV viral–viral, human–viral, human–human, kinase–viral, and kinase–human interactions. Among these, CDK9 emerged as the highest-ranked kinase with an MCC score of 2.15 and 412 connections, identifying it as the major hub kinase within the dengue-associated network.

### 2.6. Literature-Derived Host Kinase Inhibitors Relevant to DENV-2 Infection

A literature-based analysis identified potential inhibitors targeting host kinases associated with DENV-2 infection to explore their therapeutic relevance. From the compiled inhibitor data, 59 kinases identified through the Johnson et al. (2022) and Yaron-Barir et al. (2024) [25,26] motif pattern analyses and 12 kinases identified through the Poll B. G. et al. (2024) [27] motif pattern analysis were found to have reported inhibitors, highlighting their potential as antiviral targets (Supplementary Table S5). Among these, 10 kinases, cyclin-dependent kinase 2 [CDK2], epidermal growth factor receptor [EGFR], Erb-b2 receptor tyrosine kinase 2 [ERBB2], fibroblast growth factor receptor 2 [FGFR2], phosphoinositide-dependent protein kinase 1 [PDK1], CDK9, fibroblast growth factor receptor 4 [FGFR4], mitogen-activated protein kinase kinase 6 [MAP2K6], spleen tyrosine kinase [SYK], and fibroblast growth factor receptor 3 [FGFR3] were common to both datasets. Notably, CDK9, a cyclin-dependent kinase identified as a key hub kinase, was identified across motif predictions, phosphoproteomics datasets, and protein–protein interaction analyses, and also found to have inhibitor data. CDK9 was found to be inhibited using flavopiridol or microRNA [miRNA] [32]. In addition, K002 and K144 (kenpaullone) were found to have an inhibitory effect on CDKs and may prevent viral entry or genome release, thereby reducing infection [11]. Several kinases with known inhibitors were also found to be associated with DENV-2 infection, suggesting their potential relevance as therapeutic targets. These findings suggest that repurposing or optimizing existing kinase inhibitors could provide an effective strategy for developing antiviral therapies against DENV-2.

### 2.7. Docking Analysis of Predicted Host Kinase–DENV-2 Protein Interactions

To perform in silico validation of predicted interactions between host kinases and DENV-2 proteins, protein–protein docking was performed using the docking module in Maestro [33]. Predicted human kinases EEF2K, CDK9, HASPIN, and TNNI3K were docked with their corresponding viral proteins. For each interaction, 30 docking poses were generated, and the pose with the best interaction was selected for further analysis.

**Figure 4:**
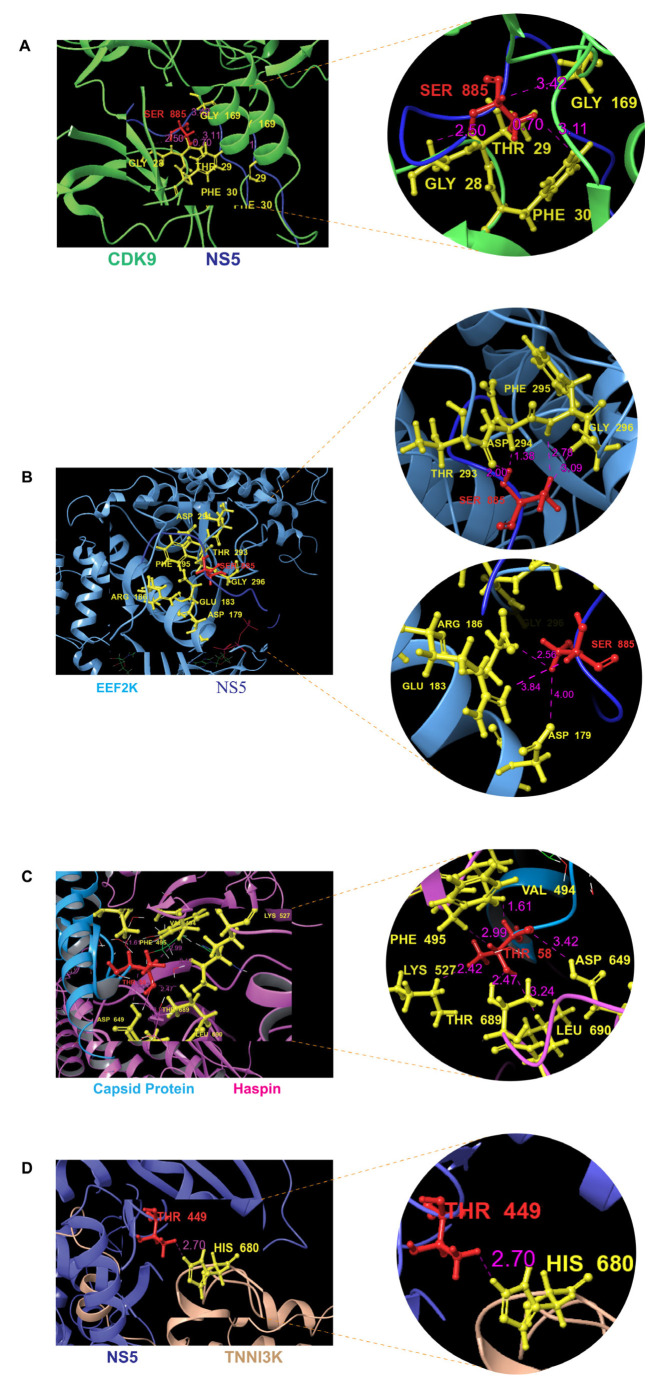
This figure illustrates the docking interactions between host kinases and viral proteins, highlighting key kinase–substrate phosphorylation sites identified in the viral protein sequences. (**A**) Interaction between human kinase CDK9 and DENV-2 protein NS5. In the figure, green indicates the kinase and blue indicates the DENV-2 protein. (**B**) Interaction between human kinase EEF2K and DENV-2 protein NS5. In the figure, light blue indicates the kinase and dark blue indicates the DENV-2 protein. (**C**) Interaction between human kinase HASPIN and DENV-2 capsid protein. In the figure, pink indicates human kinase, and blue indicates DENV-2 protein. (**D**) Interaction between human kinase TNNI3k and DENV-2 protein NS5. In the figure, peach indicates kinase, and blue indicates DENV-2 protein.

CDK9, identified as a significant kinase in both host phosphoproteomics and protein–protein interaction data, exhibited strong interaction with the DENV-2 NS5 protein (PDB ID: 3BLH and 5HHG). Pose 7 was identified as the best interaction with a PIPER pose energy of −1241.088 kcal/mol, a PIPER pose score of −48.294, and a PIPER cluster size of 51, supporting its potential role in infection (Figure 4A). Similarly, EEF2K which showed phosphosite regulation during DENV infection based on host phosphoproteomics data. Docking analysis supporting this, showing EEF2K interaction with NS5 (PDB ID: 8FNY and 5HHG), where pose 1 was identified as optimal with a PIPER pose energy of −1321.528 kcal/mol, a PIPER pose score of 3.695, and a PIPER cluster size of 195 (Figure 4B). HASPIN, which was predicted to interact with DENV-2 proteins based on protein–protein interaction data, was further validated through docking analysis, which showed that HASPIN interacts with capsid (PDB ID: 3DLZ and 6VG5), where pose 2 showed the best interaction, with a PIPER pose energy of −1416.716 kcal/mol, a PIPER pose score of −294.835, and a PIPER cluster size of 139 (Figure 4C). From the viral phosphoproteomics data, we identified T449 in the DENV-2 NS5 protein as a potential phosphorylation site. Our substrate motif-based prediction identified TNNI3K as the upstream kinase for this site. Docking confirmed TNNI3K interacts with NS5 (PDB ID: 7MGJ and 5K5M), where pose 1 was identified as the best interaction, with a PIPER pose energy of −2227.812 kcal/mol, a PIPER pose score of −209.070, and a PIPER cluster size of 106 (Figure 4D) (Supplementary Table S1).

### 2.8. Docking-Based Interaction Analysis Between Human Kinases and DENV-2 Proteins

Protein–protein interaction analysis between host kinases and the DENV-2 protein was performed based on distance, specific interactions, surface complementarity, and buried solvent-accessible surface area (SASA). Human kinase CDK9, identified as a key kinase across all our analyses, exhibited a strong interaction with the DENV-2 NS5 protein at residue S885 (Figure 4A). Five CDK9 residues, G169, E66, F30, T29, and G28, within the kinase domain were found in close proximity to S885 (NS5), indicating that through this association, these interactive interfaces may facilitate the phosphorylation process. T29 showed the closest interaction at 0.7 Å, with a surface complementarity of 0.13 and a buried SASA of 96.30% (CDK9) and 72.50% (NS5 protein). G28 interacted at 2.5 Å, with a surface complementarity of 0.3 and SASA of 73.90% (CDK9) and 72.50% (NS5 protein). F30 had an interaction distance of 3.1 Å, surface complementarity of 0.37, and SASA value of 44.50% (CDK9) and 72.50% (NS5). Notably, G169, which represents the glycine residue of the conserved DFG motif that plays a pivotal role in kinase activation and regulation, interacted at 3.2 Å with a surface complementarity of 0.37 and a SASA value of 99.30% (CDK9) and 72.50% (NS5 protein). Although E66 interacted at a relatively long distance of 4.0 Å with a surface complementarity of 0.38, it exhibited SASA values of 39.70% (CDK9) and 72.50% (NS5). There is prior evidence that the NS5 protein of DENV undergoes phosphorylation at multiple serine residues, resulting in distinct post-translational forms that influence its subcellular localization and interaction with NS3, a process essential for the formation of the viral RNA replicase complex responsible for genome synthesis [34].

The same NS5 residue, S885, was also found to interact with EEF2K kinase domain residues D179, E183, R186, and T293, including the conserved DFG motif residues D294, F295, and G296 (Figure 4B), in which D294 formed the closest contact at 1.8 Å with a high surface complementarity of 0.73 and SASA values of 88.90% (EEF2K) and 75.50% (NS5). F295 interacted at 2.7 Å, with a surface complementarity of 0.50 and SASA values of 86.00% (EEF2K) and 75.50% (NS5). G296 interacted at a 3.1 Å distance, with surface complementarity of 0.73 and SASA values of 85.70% (EEF2K) and 75.50% (NS5). Among the other residues, T293 showed a strong interaction with S885 at 2.0 Å, forming one hydrogen bond with a surface complementarity of 0.76 and SASA values of 52.20% (EEF2K) and 75.50% (NS5). At the same time, E183 interacted at 2.5 Å, with a surface complementarity of 0.77 and SASA values of 91.90% (EEF2K) and 75.50% (NS5). R186 interacted at a distance of 3.4 Å with a surface complementarity of 0.72 and SASA values of 57.00% (EEF2K) and 75.50% (NS5). D179 interacted at a longer distance of 4.0 Å, had a surface complementarity of 0.77, and SASA values of 75.30% (EEF2K) and 75.50% (NS5).

HASPIN kinase domain residues L690, T689, D649, K527, F495, and V494 were positioned in close proximity to residue T58 on the capsid. (Figure 4C). Notably, the T689 residue of the conserved DYT motif of HASPIN represented the most functionally significant contact, forming a stabilizing hydrogen bond with T58 at 2.5 Å, with a surface complementarity of 0.59 and SASA values of 96.70% (HASPIN) and 87.70% (capsid). The D649 residue of the conserved DFG motif of HASPIN showed an interaction at a 3.4 Å distance, with surface complementarity of 0.48 and SASA values of 100% (HASPIN) and 87.70% (capsid). K527 interacted at 2.4 Å distance with a surface complementarity of 0.53 and SASA values of 38.90% (HASPIN) and 87.70% (Capsid). V494 formed the closest contact at 1.6 Å, resulting in two van der Waals clashes with a surface complementarity of 0.44 and SASA values of 93.50% (HASPIN) and 87.7% (capsid). L690 interacted at 3.2 Å with a surface complementarity of 0.53 and SASA values of 82.00% (HASPIN) and 87.70% (capsid). F495 interacted at 3.0 Å with a surface complementarity of 0.42 and SASA values of 71.80% (HASPIN) and 87.70% (Capsid).

H680 in the kinase domain residue of TNNI3K interacted with experimentally predicted site T449 of NS5 at a distance of 2.7 Å, with a surface complementarity of 0.77 and SASA values 86.20% (TNNI3K) and 44.40% (NS5) (Figure 4D) (Supplementary Table S1).

### 2.9. Molecular Dynamics Simulation

#### 2.9.1. Protein Root-Mean-Square Deviation (RMSD)

The RMSD of the protein backbone was calculated using the Cα atoms, with each trajectory frame aligned to the initial reference structure. RMSD values were computed as the average positional deviation of the Cα atoms in each frame relative to the reference frame, providing insight into the overall structural stability of the protein during the simulation.

The protein RMSD exhibited a small initial increase during the first few nanoseconds of the simulation. Following this initial adjustment phase, the RMSD values stabilized and converged at around ~3.5 Å, indicating that the protein attained a stable conformational state and maintained structural integrity throughout the remainder of the simulation. The absence of large fluctuations or progressive drift in the RMSD suggests that the system was well-equilibrated and did not undergo any major conformational changes during the production run.

#### 2.9.2. Protein–Protein Interactions

Protein–peptide interactions were analyzed at the interface of the complex throughout the molecular dynamics simulation (face-to-face displaced, face-to-face, and edge-to-face), water-mediated bridges, hydrophobic contacts, and aromatic hydrogen bonds (hbond_aromatic_sb, hbond_aromatic_self, and hbond_aromatic_ss).

Molecular dynamics simulations of the protein–peptide complex (chain A: protein; chain B: peptide) revealed a stable interaction interface maintained throughout the trajectory. Interaction occupancies were calculated over the full simulation, and only contacts persisting for more than 10% of the simulation time were considered. Hydrogen bonding interactions played a major role in stabilizing the peptide anchoring. A stable aromatic sidechain hydrogen bond between A:CYS106 and B:TYR882 was observed with an occupancy of 52.4%. Backbone hydrogen bonds involving A:GLU57-B:ARG891 (37.1%) and A:GLU55-B:ARG891 (26.2%) further stabilized the peptide backbone, while A:GLU57-B:GLU893 contributed with 13.5% occupancy. Highly persistent sidechain–sidechain hydrogen bonds were detected between A:GLU66-B:ASP881 (85.1%) and A:GLU66-B:SER885 (80.9%), identifying this region as a major hydrogen-bonding hotspot. Notably, the A:GLU66-B:SER885 interaction, which was identified in the docking analysis, further supports the stability of the complex.

**Figure 5:**
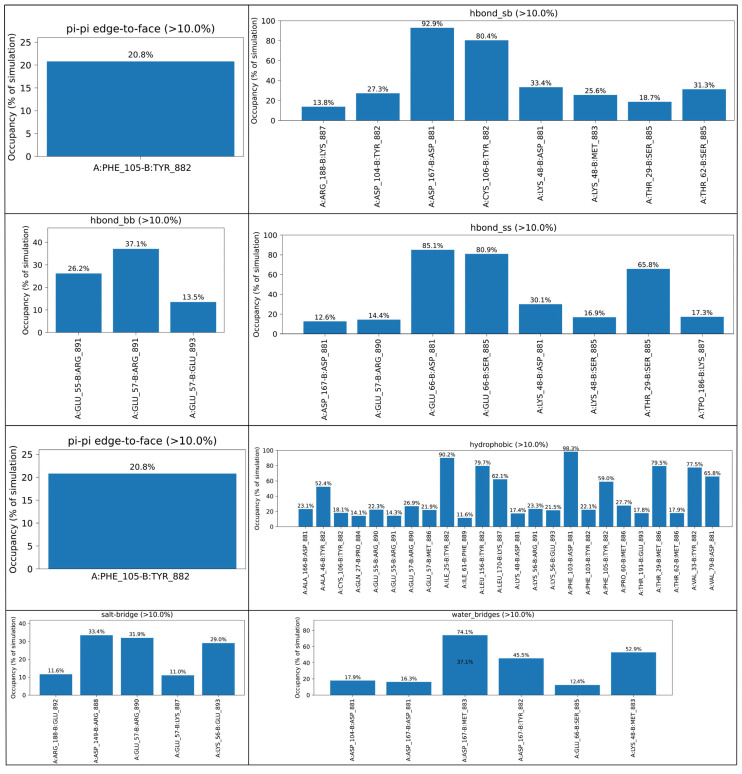
Protein–protein interaction during MD simulation.

Electrostatic stabilization was reinforced by salt bridges, particularly A:ASP149-B:ARG888 (33.4%), A:GLU57-B:ARG890 (31.9%), and A:LYS56-B:GLU893 (29.0%). Hydrophobic interactions formed the dominant interaction class, with very high occupancies observed for A:PHE103-B:ASP881 (98.3%), A:ILE25-B:TYR882 (90.2%), A:LEU156-B:TYR882 (79.7%), and A:THR29-B:MET886 (79.5%), indicating tight packing at the interface. An aromatic π–π edge-to-face interaction between A:PHE105 and B:TYR882 was present for 20.8% of the simulation. Water-mediated interactions further stabilized the complex, with persistent water bridges involving A:ASP167-B:MET883 (74.1%), A:LYS48-B:MET883 (52.9%), and A:ASP167-B:TYR882 (45.5%) (Figure 5). Collectively, these interactions indicate a robust and well-organized protein–peptide binding interface.

### 2.10. Conservation of Structurally Validated Sites in DENV Proteins

**Figure 6:**
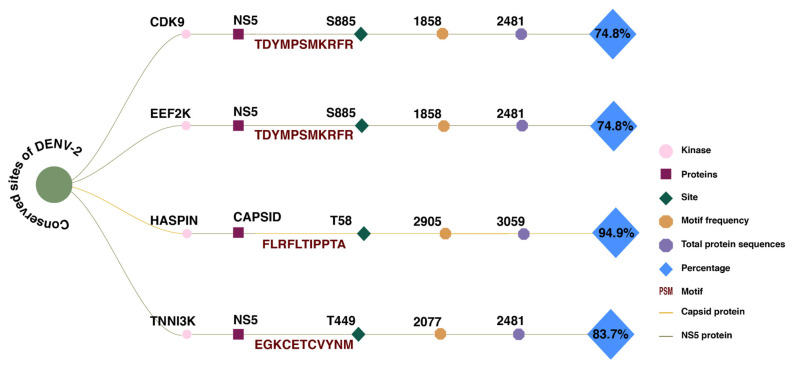
Conservation of docked phosphorylation sites in DENV-2 proteins and their predicted upstream kinases.

The predicted phosphorylation sites of DENV-2 proteins that were docked and identified to interact with human kinases were found to be conserved across variants. The DENV-2 protein NS5 motif TDYMPSMKRFR (S885), identified as a potential phosphorylation site for both CDK9 and EEF2K, showed 74.8% conservation. The DENV-2 protein capsid motif FLRFLTIPPTA (T58), associated with HASPIN, showed the highest level of conservation at 94.9%. The experimentally validated DENV-2 NS5 motif, EGKCETCVYNM (T449), which was predicted to interact with TNNI3K, showed 83.7% conservation. The conservation of these sites suggests their potential role in the infection and host–virus interactions (Figure 6; Table 2).

### 2.11. Assessment of Motif-Based Phosphorylation Predictions Using Computational Tools

**Figure 7:**
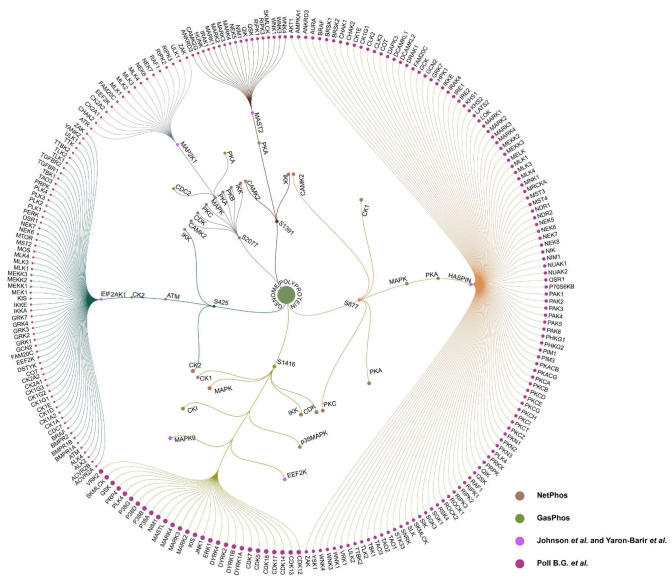
Circular raw graph of commonly predicted phosphorylation sites in the dengue genome polyprotein. The inner node and stroke color represent the sites.

Computational predictions using NetPhos 3.1 and GasPhos were performed on the DENV-2 protein sequences selected after docking analysis (Supplementary Table S6). These predictions identified the same phosphorylation sites as those obtained from the studies by Johnson et al. (2022) and Yaron-Barir et al. (2024) [25,26], and Poll B. G. et al.’s kinase–substrate motif analysis (Figure 7; Supplementary Table S6), thereby reinforcing the reliability of docking-based validation. The S885 residue of the NS5 protein, identified as a phosphorylation site through the Poll B. G. et al. (2024) [27] motif-based pattern analysis and docked with the upstream kinases CDK9 and EEF2K, was also predicted as a phosphorylation site by both the NetPhos 3.1 and GasPhos (version 2020) computational tools, reinforcing the reliability of the prediction. GasPhos predicted kinases belonging to the CDK and PKC families, whereas NetPhos predicted the PKC family. In addition to CDK9 and EEF2K, the Poll B. G. et al. (2024) [27] motif pattern also predicted multiple kinases for S885, including Cdc-like kinase 2 [CLK2], CDC-like kinase 3 [CLK3], dual-specificity tyrosine-phosphorylation-regulated kinase 1A [DYRK1A], mitogen-activated protein kinase 9 [MAPK9], mitogen-activated protein kinase kinase 1 [MAP2K1], and mitogen-activated protein kinase kinase 7 [MAP2K7]. Similarly, the T58 of the capsid protein, predicted to be phosphorylated by HASPIN based on the Poll B. G. et al. (2024) [27] motif, was also predicted by both prediction tools to be targeted by PKA family kinases.

Comparative analysis integrating the motif patterns predictions with computational predictions from NetPhos 3.1 and GasPhos revealed several commonly predicted phosphorylation sites, including S667 and S425 of the envelope protein, S2077 of NS3, and S1416 and S1393 of NS2B. Notably, while motif-based analyses predict specific kinases, NetPhos and GasPhos predict kinase families for the particular sites. Across all predictions, kinases belonging to the CDK, MAPK, PKC, PKA, CK1, IKK, and CAMK families were the most frequently identified, suggesting their potential roles as key regulatory kinases in DENV-2 protein phosphorylation.

## 3. Discussion

**Figure 8:**
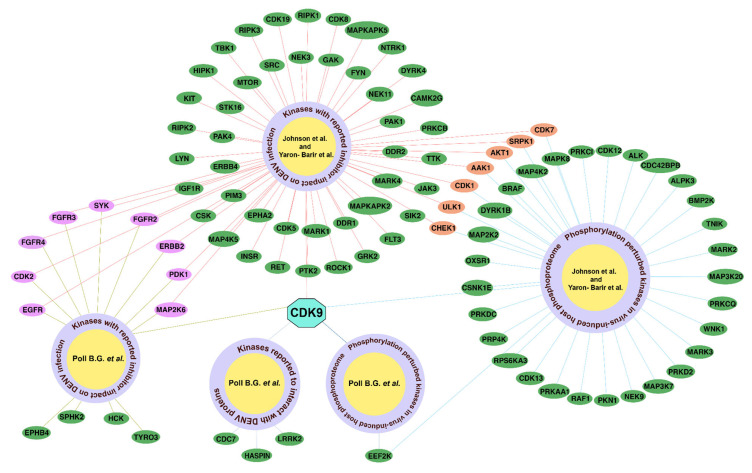
Pictorial representation showing CDK9 as the hub kinase identified through all comparative analyses.

Dengue is emerging as the second most serious vector-borne viral infection, with its rapid spread causing significant illness and death worldwide [35], underscoring the urgent need for effective antiviral strategies [12,13,14,15,16]. Many positive-sense RNA viruses manipulate host cellular membranes to facilitate viral replication [36]. To mitigate this, we sought to identify host regulators of DENV-2 infection to uncover therapeutic interventions. Phosphorylation mediated by protein kinases is a critical post-translational modification in host cells, essential for many intracellular pathogens to establish a productive infection cycle. Protein phosphorylation is necessary for the proper functioning of many RNA viruses, including Flaviviruses [37]. So, we chose to focus on host kinases in our study, as they play crucial roles in regulating viral infection processes and may act as potential therapeutic targets for DENV-2. We combined motif-based phosphorylation site prediction with host–virus phosphoproteomic and protein–protein interaction analyses to identify potential host kinases targeting DENV-2 proteins. This was further reinforced with computational kinase prediction tools and molecular docking. Since molecular docking is based on static protein structures and may not fully capture conformational flexibility, we performed a molecular dynamics simulation for one selected kinase–viral protein complex to illustrate the stability of the docked interactions over time. Considering that one kinase can phosphorylate multiple viral proteins/phosphosites and also that one viral protein/phosphosite could be the target of multiple kinases (kinase redundancy), we set out to identify a major hub kinase in this network as the most considerable target of therapeutics. Across our analyses, CDK9 emerged as a hub kinase, identified through both the pattern analysis in host phosphoproteomics and protein–protein interaction data, and through known pharmacological inhibitors (Figure 8). Quantitative hub analysis using the MCC algorithm in CytoHubba further confirmed CDK9 as the hub kinase within the integrated dengue-associated interaction network, providing network-based support for its central regulatory role. Prior studies have shown that CDK9 plays a key role in DENV-2 infection by forming the P-TEFb complex with cyclin T1, which facilitates virus-induced transcription. Its interaction with the DENV capsid protein and P-TEFb enhances NF-κB activation and IL-8 expression, resulting in host inflammatory response. Inhibition of CDK9 with DRB or silencing of cyclin T1 with siRNA has been shown to suppress IL-8 induction in infected cells, confirming the essential role of P-TEFb in this process. Flavopiridol binds directly to the ATP-binding pocket of CDK9, thereby blocking substrate phosphorylation and downstream transcriptional activation. These findings suggest that targeting CDK9 with selective inhibitors like flavopiridol or using miRNAs to downregulate cyclin T1 may serve as potential therapeutic strategies to reduce cytokine-driven inflammation in severe dengue [3,32,38]. Disrupting the transcriptional machinery hijacked by the virus may, therefore, represent a promising strategy to control both viral replication and host inflammatory damage.

Our motif pattern analysis revealed that NS5 is the most frequently targeted DENV protein by host kinases. NS5 is the largest of the ten DENV proteins and serves as the central enzyme for viral RNA replication, with both RNA-dependent RNA polymerase and methyltransferase functions. Notably, two experimentally validated phosphorylation sites during DENV infection were also identified within NS5. Among these, threonine 449 (T449) is a conserved phosphorylation site, for which we predicted TNNI3K as the upstream kinase. In contrast, the phosphorylation at T449 plays a regulatory role in the virus’s replication cycle [30]. Targeting kinases responsible for NS5 phosphorylation may, therefore, represent a promising therapeutic strategy. Supporting this, prior studies have shown that phosphorylation of the experimentally validated site T395 of the DENV NS5 protein by the host kinase CK2 inhibits its nuclear localization, thereby inhibiting transcription [29]. Based on this, we hypothesize that phosphorylation of NS5 at T395 by other host kinases may induce NS5 activity, influencing viral replication and immune evasion. Eleven kinases predicted through our motif-based analysis were found to interact with the NS5 protein, confirming their association in protein–protein interaction data. Among these, three kinases were identified to have inhibitory data. Activation of mTOR triggers mTORC1 signaling, which induces lipophagy to support DENV replication. Inhibition of mTOR using rapamycin disrupts autophagic flux and significantly reduces viral replication [39]. SB203580 is a selective inhibitor that binds competitively to the ATP-binding pocket of p38 mitogen-activated protein kinase [p38 MAPK], thereby blocking its catalytic activity and preventing phosphorylation of downstream targets such as MAPK-activated protein kinase 2 [MAPKAPK2] [40]. Inhibition of SRPK1 using SFV785 impairs viral assembly by disrupting host kinase activity [41].

Inhibitor of nuclear factor kappa-B kinase subunit epsilon [IKBKE] identified from our motif pattern analysis (Johnson et al. (2022), Yaron-Barir et al. (2024) and Poll B. G. et al. (2024)) [25,26,27] has been reported in protein–protein interaction data to interact with several DENV viral proteins, including NS2B and NS3. Previous studies have shown that during DENV infection, the NS2B/NS3 protein binds to IKBKE and masks its ATP-binding domain, thereby inhibiting its kinase activity. IKBKE phosphorylates IRF3, which, in turn, activates IFN-β and initiates an antiviral response. However, by blocking the kinase domain of IKBKE, the NS2B/NS3 protein disrupts IRF3 phosphorylation, suppressing the host antiviral signaling pathway and facilitating viral immune evasion [42]. Based on these findings, we hypothesize that the interaction between IKBKE and the dengue proteins NS2B/NS3 involves a phosphorylation-mediated regulatory mechanism, in which IKBKE phosphorylation contributes to immune suppression and enhances viral pathogenesis. Taken together, all our findings suggest that targeting host kinases may represent a potential host-directed therapeutic strategy against DENV-2. However, further experimental validation, such as kinase inhibition or knockdown assays to assess host kinase dependence, site-directed mutagenesis of predicted viral phosphorylation sites to evaluate their functional relevance, and co-immunoprecipitation or pull-down assays to confirm kinase–viral protein interactions, is required to substantiate these findings and explore their potential clinical relevance.

## 4. Materials and Methods

### 4.1. Selection of Kinase–Substrate Phosphomotifs

Johnson et al. (2022) mapped kinase–substrate interaction patterns across the human kinome by defining the substrate specificities of 303 serine/threonine kinases using synthetic peptide libraries and positional scanning [25]. Similarly, Yaron-Barir et al. (2024) characterized 78 catalytically active human tyrosine kinases using positional-scanning peptide arrays and identified distinct sequence preferences around phosphotyrosine sites, including phosphopriming effects and charge-based selectivity, which grouped tyrosine kinases into functional clusters [26]. From these two datasets, we extracted kinase-specific sequence motifs for serine/threonine and tyrosine kinases with percentile scores above 85, focusing on enriched amino acid preferences surrounding the phosphorylation sites for our analysis.

Sugiyama N. et al. (2019) discovered phosphorylation patterns for 385 recombinant human protein kinases over 175,574 potential direct substrates using large-scale mass spectrometry and generated a collection of kinase–substrate sequence preference motifs for each kinase [24]. Building on this dataset, Poll B. G. et al. (2024) [27] generated 13-amino-acid sequence motif logos using the PTM-Logo software (version 2019) to represent substrate preferences for 384 recombinant human protein kinases visually. A minimum of 30 target amino acid sequences was required to generate a kinase logo, although this threshold could vary depending on the strength of kinase–substrate interactions [27]. From this dataset, we selected 330 statistically significant human kinase–substrate sequence motifs for our analysis.

### 4.2. Phosphomotif-Based Prediction of Human Kinase Targets in DENV Proteins

FASTA sequences of DENV [taxonomy ID: 12637] proteins were retrieved from UniProt [43]. The kinase–substrate phosphomotif sequences obtained from the studies by Johnson et al. (2022), Yaron-Barir et al. (2024) and Poll B.G. et al. (2024) [25,26,27] were searched against these DENV protein sequences to identify potential human kinases capable of phosphorylating DENV proteins. The matched protein sequences were then mapped to a reference DENV genome polyprotein [UniProt ID: P29991] [44] to define the range of each of the ten viral proteins. Each predicted phosphorylation site was assigned to its corresponding viral protein based on its mapped position. Although motif pattern searches were carried out across all four DENV serotypes, further analyses focused on DENV-2, as it is considered one of the most virulent serotypes, with more severe illness and larger outbreaks [45,46].

### 4.3. Phosphoproteomic Analysis of Host Kinase and DENV Phosphorylation Sites During DENV-2 Infection

To investigate whether host kinases associated with DENV-2 infection have already been identified, we searched the literature on PubMed using the query “phosphoproteomics” AND “Dengue.” From this search, we retrieved a differential phosphoproteomics dataset specific to DENV-2 infection versus uninfected conditions, based on statistical significance (standard deviation (SD) > 1.96 and *p*-value < 0.05) [28]. To ensure uniform annotation, each phosphosite in the dataset was mapped to its corresponding UniProt accession (retrieved in February 2025) using a custom-built mapping tool based on UniProt 2023 [43] standards. Additionally, the regulatory information of identified phosphosites was analyzed using the curated regulatory site information from the PhosphoSitePlus database [47].

To investigate whether the predicted phosphorylation sites in DENV-2 proteins have been previously reported, we searched available viral phosphoproteomics data. Experimentally validated phosphorylation sites were retrieved from the Viral Posttranslational Modification Database [VPTMdb] [31].

### 4.4. Integrative Analysis of Human–Viral Protein Interactions in DENV-2 Infection

To identify potential interactions between predicted human kinases and DENV-2 proteins, we retrieved previously reported protein–protein interaction [PPI] data related to DENV from multiple publicly available databases, including the Human–Virus Interaction Database [HVIDB] [48], the Human–Virus Protein–Protein Interaction Database [HVPPI] [49], the Virus–Host Network [VirHostNet3.0] [50], the Host–Pathogen Interaction Database [HPIDB 2.0] [51], the Molecular Interaction Database [IntAct] [52], and the Dengue–Human Interaction Database [DenHunt] [53].

### 4.5. Hub Analysis

Hub analysis was performed in Cytoscape (v3.10.3) [54] using the CytoHubba plugin to identify major hub kinases. The Maximal Clique Centrality (MCC) algorithm was applied to evaluate kinase centrality within the integrated dengue-associated interaction network. The network was constructed using DENV viral–viral protein interactions, human–viral protein interactions, human–human protein interactions, and interactions of host kinases predicted from both studies by Johnson et al. (2022) and Yaron-Barir et al. (2024) and the Poll B. G. et al. (2024) [25,26,27] motif pattern analysis that overlapped with total kinases in proteomics and phosphoproteomics perturbed in dengue infection, with DENV proteins and human proteins.

### 4.6. Exploring Known Kinase Targets and Their Regulatory Roles in DENV-2 Infection

To assess the therapeutic potential of kinases predicted by kinase–substrate motif analysis, we conducted a PubMed search using the query “Kinase target” AND “DENV-2”, aiming to identify known inhibitors with reported effects on DENV-2 infection. The retrieved data were analyzed to elucidate the regulatory roles of these kinases during infection and to evaluate how their inhibition influences kinase activity and viral replication.

### 4.7. Structural Interaction Analysis of Human Kinases and DENV-2 Proteins via Molecular Docking

To analyze the structural interaction between predicted human kinases and their corresponding phosphorylated DENV-2 proteins, we obtained 3D structures from the Protein Data Bank [PDB] based on resolution and structural relevance. Docking was performed for kinases identified as potential regulators from phosphoproteomic and protein–protein interaction data, as well as those associated with experimentally validated phosphorylation sites on the DENV-2 protein during infection. The corresponding DENV-2 proteins for each kinase were chosen based on the availability of structures with the predicted phosphorylation regions modeled. Since most of the phosphorylation sites predicted using the Johnson et al. (2022) and Yaron-Barir et al. (2024) [25,26] motif patterns were not modelled, and each protein generally contained only a single site, the motif prediction results from Poll B. G. et al. (2024) [27] were used for structural validation. The 3D structures of human kinases and corresponding DENV proteins were obtained from the PDB database, with their PDB IDs and resolutions provided in Supplementary Table S1. All protein structures were optimized and energy-minimized using the Protein Preparation Wizard with the OPLS4 force field in Schrödinger (version 2025-4).

Protein–protein docking between selected human kinases and DENV protein was performed using the BioLuminate module in Maestro v14.6 [33] to evaluate whether the identified phosphorylation sites in viral protein interact with their corresponding kinase (Shaji, Rafi, et al., 2025) [55] (Shaji, Anil, et al., 2025) [12]. DENV-2 proteins were taken as ligands and human kinases as receptors. The DENV-2 capsid protein was docked with human kinase HASPIN, NS1 was docked with EEF2K, and NS5 was docked with CDK9, EEF2K, and TNNI3K. The Protein Interaction Analysis module in Maestro v14.6 was used to analyze the docked protein–ligand complex. For each complex, 30 docking poses were generated based on PIPER cluster size, PIPER pose energy, and PIPER pose score, evaluating receptor–ligand interactions and computed using Fast Fourier Transforms [56]. To assess whether the interacting phosphorylation sites were conserved, we checked the conservation of the identified DENV-2 phosphosites across corresponding viral variant sequences.

### 4.8. MD Simulation

Molecular dynamics (MD) simulations were performed using the Desmond module within the Schrödinger suite. The docking complex between CDK9 and the DENV-2 NS5 protein, along with the key residue interactions, was generated using the protein–protein docking workflow in BioLuminate (Schrödinger Maestro 2025-3; Schrödinger, LLC, New York, NY, USA). Protein preparation involved the addition of missing hydrogen atoms, assignment of correct bond orders, optimization of protonation states, and restrained energy minimization. All simulations were carried out using the OPLS4 force field. The prepared protein was placed in an orthorhombic simulation box with a 10 Å buffer distance from the box boundaries and solvated using the SPC water model. The system was neutralized by the addition of five chloride (Cl^−^) ions, followed by the addition of NaCl to maintain a physiological salt concentration of 0.15 M. The system was subjected to the default Desmond minimization and equilibration protocol, after which a 100 ns production MD simulation was performed. Trajectory frames were recorded at 100 ps intervals for subsequent structural and dynamic analyses. The root-mean-square deviation (RMSD) and protein–protein interaction analysis were used to understand the stability of the complex

### 4.9. Characterization of Predicted Phosphorylation Sites via Kinase Prediction Tools

To further substantiate our motif-based predictions, we used computational kinase prediction tools, such as NetPhos 3.1 and GasPhos, to determine whether they could identify the same DENV phosphorylation sites and their corresponding kinases. The DENV-2 protein sequences containing the docked phosphorylation sites were used as input for these tools. In addition, we performed a comparative analysis integrating predictions from all approaches (NetPhos, GasPhos, Johnson et al. (2022) and Yaron-Barir et al. (2024), and Poll B.G. et al.’s (2024) [25,26,27] motif pattern) to identify overlapping or commonly predicted phosphorylation sites other than the docked site.

## 5. Conclusions

In conclusion, the dengue virus remains a formidable challenge for the development of effective vaccines and therapeutics. Our findings emphasize that DENV exploits host kinases to manipulate cellular signaling pathways, promoting its replication and survival. A deeper understanding of these virus–host kinase interactions offers critical insights into the mechanisms by which DENV modulates host cellular processes, paving the way for the development of targeted antiviral strategies. Our integrative computational analysis identified several host kinases capable of phosphorylating DENV-2 proteins, with CDK9 emerging as the most significant candidate. Docking analyses further revealed strong interactions between DENV-2 proteins and kinases, including EEF2K, HASPIN, and TNNI3K. Targeting CDK9-associated pathways may, therefore, represent a promising approach for antiviral drug development. Since many of the identified kinases already have known pharmacological inhibitors, these findings provide a strong rationale for exploring host-directed antiviral strategies that focus on kinase-mediated regulation. Experimental validation of these predicted kinase–viral protein interactions, along with kinase inhibition studies, will be essential to confirm their functional relevance and therapeutic potential. Collectively, these findings provide mechanistic insights into kinase-driven host–virus signaling mechanisms and underscore the central role of phosphorylation in dengue pathogenesis and antiviral drug development.

## Figures and Tables

**Table 1:** Summary of human kinases predicted using kinase–substrate motif analyses that have been previously reported to interact with DENV-2 viral proteins.

Kinase–Substrate Motif Studies	DENV-2 Viral Proteins	Kinases
Poll B. G. et al.	Capsid	LRRK2, CDK9, HASPIN
Poll B. G. et al.	NS1	LRRK2
Poll B. G. et al.	NS3	CDC7, LRRK2
Poll B. G. et al.	NS5	HASPIN
Johnson et al., Yaron-Barir et al.	Capsid	PRKDC, MTOR, RAF1, SRPK1, CDK1, ATR, LRRK2, CAMK2B, MAPKAPK5, EIF2AK2, ZAP70, CSNK2A1, CSNK1A1, MAPKAPK2, SRPK2, PRP4K, HASPIN, PRKCE
Johnson et al., Yaron-Barir et al.	Envelope	MTOR, RAF1, SRPK1, ZAP70, CSNK2A1, PRKDC, ATR, LRRK2, MAPKAPK5, EIF2AK2
Johnson et al., Yaron-Barir et al.	Membrane	PRKDC, ATR, LRRK2, MAPKAPK5, EIF2AK2
Johnson et al., Yaron-Barir et al.	NS1	AKT1, GSK3B, CHEK1, SIK3, IRAK1, CDK1, MTOR, RAF1, CSNK2A1, PRKDC, LRRK2, MAPKAPK5, EIF2AK2
Johnson et al., Yaron-Barir et al.	NS2A	CLK1, IKBKE, TBK1, MAPK1, STK39, CAMK2B, IRAK1, CDK1, MTOR, RAF1, SRPK1, ATR, CSNK2A1, PRKDC, LRRK2, MAPKAPK5, EIF2AK2
Johnson et al., Yaron-Barir et al.	NS2B	MAP2K2, PAK1, CAMK2D, TTBK2, IKBKE, CAMK2B, IRAK1, CDK1, MTOR, ATR, PRKDC, LRRK2, EIF2AK2
Johnson et al., Yaron-Barir et al.	NS3	CDC7, PRKACA, CDK18, STK3, CAMK2G, PKN2, BMP2K, CAMK2D, SRPK1, IKBKE, CAMK2B, IRAK1, RAF1, CDK1, CSNK2A1, MTOR, ATR, MAPKAPK5, PRKDC, LRRK2, EIF2AK2
Johnson et al., Yaron-Barir et al.	NS4A	VRK2, TBK1, MAP2K2, CDK18, ZAP70, IKBKE, RAF1, CDK1, MTOR, ATR, MAPKAPK5, LRRK2, EIF2AK2
Johnson et al., Yaron-Barir et al.	NS4B	CLK1, TBK1, ZAP70, IRAK1, IKBKE, CSNK2A1, RAF1, CDK1, PRKDC, ATR
Johnson et al., Yaron-Barir et al.	NS5	LTK, CSNK1D, CSNK1A1, MAPKAPK2, SRPK2, PRP4K, HASPIN, PAK1, SRPK1, CAMK2B, CSNK2A1, MTOR, RAF1, PRKDC, ATR, LRRK2, EIF2AK2

**Table 2:** Conservation of structurally validated phosphorylation sites in DENV-2 proteins.

Kinases	Mode_Sequence	Site	Protein	Mode_Frequency	Total_Sequence	% Conservation
CDK9	TDYMPSMKRFR	S885	NS5	1858	2481	74.8891576
EEF2K	TDYMPSMKRFR	S885	NS5	1858	2481	74.8891576
HASPIN	FLRFLTIPPTA	T58	Capsid	2905	3059	94.96567506
TNNI3K	EGKCETCVYNM	T449	NS5	2077	2481	83.71624345

## Data Availability

The original contributions presented in this study are included in the article/Supplementary Material. Further inquiries can be directed to the corresponding author.

## Supplementary Materials

The following supporting information can be downloaded at https://www.mdpi.com/article/10.3390/ijms27062718/s1.

## Abbreviations

DENV	Dengue virus
DENV-2	Dengue virus type 2
C	Capsid protein
prM/M	Precursor membrane/membrane protein
E	Envelope protein
NS	Non-structural protein
WHO	World Health Organization
MAPK	Mitogen-activated protein kinase
JNK	c-Jun N-terminal kinase
NTRK1	Neurotrophic receptor tyrosine kinase 1
MAPKAPK5	MAP kinase-activated protein kinase 5
HIV	Human immunodeficiency virus
SD	Standard deviation
VPTMdb	Viral Posttranslational Modification Database
PPI	Protein–protein interaction
HVIDB	Human–Virus Interaction Database
HVPPI	Human–Virus Protein–Protein Interaction Database
VirHostNet3.0	Virus–Host Network
HPIDB	Host–Pathogen Interaction Database
IntAct	Molecular Interaction Database
DenHunt	Dengue–Human Interaction Database
PDB	Protein Data Bank
MD	Molecular dynamics
HASPIN	Haploid germ cell-specific nuclear protein kinase
EEF2K	Eukaryotic elongation factor 2 kinase
CDK9	Cyclin-dependent kinase 9
TNNI3K	Troponin-I-interacting kinase
MAP3K7	Mitogen-activated protein kinase kinase kinase 7
MARK3	Microtubule affinity-regulating kinase 3
PRKDC	DNA-dependent protein kinase catalytic subunit
PRKCQ	Protein kinase C theta
MARK2	Microtubule affinity-regulating kinase 2
CDK1	Cyclin-dependent kinase 1
CDK13	Cyclin-dependent kinase 13
ALPK3	α protein kinase 3
CDK12	Cyclin-dependent kinase 12
MAP2K2	Mitogen-activated protein kinase kinase 2
CK2	Casein kinase 2
PKG	cGMP-dependent protein kinase
ACVR1	Activin receptor type I
BMPR1A	Bone morphogenetic protein receptor type-1A
MAST2	Microtubule-associated serine/threonine kinase 2
PRP4K	Pre-mRNA-processing factor 4 kinase
CHEK1	Checkpoint kinase 1
RAF1	RAF proto-oncogene serine/threonine-protein kinase
AKT1	RAC-alpha serine/threonine-protein kinase
SRPK1	Serine/arginine-rich protein kinase-1
BMP2K	Bone morphology protein 2-inducible kinase
LRRK2	Leucine-rich repeat kinase 2
CDC7	Cell division cycle 7
CDK2	Cyclin-dependent kinase 2
EGFR	Epidermal growth factor receptor
ERBB2	Erb-b2 receptor tyrosine kinase 2
FGFR2	Fibroblast growth factor receptor 2
PDK1	Phosphoinositide-dependent protein kinase 1
FGFR4	Fibroblast growth factor receptor 4
MAP2K6	Mitogen-activated protein kinase kinase 6
SYK	Spleen tyrosine kinase
FGFR3	Fibroblast growth factor receptor 3
miRNA	microRNA
SASA	Solvent-accessible surface area
RMSD	Root-mean-square deviation
CLK2	Cdc-like kinase 2
CLK3	CDC-like kinase 3
DYRK1A	Dual-specificity tyrosine-phosphorylation-regulated kinase 1A
MAPK9	Mitogen-activated protein kinase 9
MAP2K1	Mitogen-activated protein kinase kinase 1
MAP2K7	Mitogen-activated protein kinase kinase 7
p38 MAPK	p38 mitogen-activated protein kinase
MAPKAPK2	MAPK-activated protein kinase 2
IKBKE	Inhibitor of nuclear factor kappa-B kinase subunit epsilon
